# From dust to flesh: mapping the path of restorative justice through a transcorporeal reading of Nguyễn Phan Quế Mai’s *Dust Child*

**DOI:** 10.3389/fsoc.2026.1816441

**Published:** 2026-06-16

**Authors:** J. Jeni Jovitha, Daisy Gohain

**Affiliations:** Vellore Institute of Technology, Chennai, India

**Keywords:** Agent Orange, Amerasians, *Dust Child*, Nguyễn Phan Quế Mai, restorative justice, slow violence, testimonial witnessing, Transcorporeality

## Abstract

Nguyễn Phan Quế Mai’s *Dust Child* (2023) provides a transgenerational narrative of the Vietnam civil war repercussions in the context of restorative justice, offering a critical discussion of how first-person accounts serve as a sociological tool for storytelling, testimony, and reconciliation within post-war Vietnam. Methodologically situated within a sociology of literature framework, this research article utilizes a multi-layered theoretical approach combining Rob Nixon’s Slow Violence, Stacy Alaimo’s Transcorporeality, and John Braithwaite’s Restorative Justice, supplemented by frameworks of Trauma and Testimonial Witnessing. Through this lens, the study evaluates the bodily trauma of Phong, an Amerasian child, Dan, an American Veteran, and war-survivors Trang and Quỳnh, revealing how the novel depicts embodied and ecological trauma as a site of collective social and moral accountability. The textual results demonstrate that slow violence persists across bodies, landscapes, and generations while actively challenging the historical silence surrounding chemical warfare. Ultimately, this article discusses how narrating intergenerational trauma through intimate personal testimonies positions storytelling as a vital sociological process of acknowledgment, ethical witnessing, and restorative reconciliation in contemporary post-war Vietnam.

## 1 Introduction

War is a catastrophic event that creates irreversible damage and destruction to humans and nature. This event is often highlighted by immediate devastation, chaos, and loss of human lives. The delayed and incremental effects of violence are frequently archived within the environment and human bodies. As Buell (1995) states, “nature is not a passive background but an active element that is being destroyed and decapitated.” The central idea of this study is that the most destructive consequences of war are not always immediate. It inflicts Slow Violence over time, resisting visibility and closure. Additionally, the social structure is disrupted by anger, frustration, and hate. In *Dust Child* (2023) by Nguyễn Phan Quế Mai, the environmental and bodily containment of Agent Orange serves as a physical testimony to depict the aftermath of chemical warfare. Delayed harm has been ignored, and justice is yet to be served through standard courtroom procedures. The study identifies this central problem as a gap in our communal and legal paradigms. Hence, Restorative Justice is advocated as a sociological framework that connects war-torn communities in international attempts to achieve peace and reconciliation. This research reveals the toxic archive of Agent Orange by examining the transcorporeal effects, past suffering, and trauma of both communities. This study contends that because the Slow Violence inflicted on humanity and the environment remains invisible, thereby evading judicial procedures and immediate litigation, the interpretation of *Dust Child* serves as a crucial framework for Restorative Justice. This is achieved through collective healing and witnessing in a society that shares a common history of violence, with a new foundation for peace.

Existing scholarly studies on Agent Orange widely engage with empirical, toxicological, and epidemiological frameworks. These empirical studies quantify toxicity without fully understanding the ecological, cultural, ethical, and historical implications. In addition, the framework of Transcorporeality has been applied to climate discourse and speculative fiction, but its application to historical and chemical warfare remains limited. Despite its relevance, Nguyễn Phan Quế Mai’s *Dust Child* has received limited attention in the eco-literary and sociological analyses. This study addresses this void by synthesizing Rob Nixon’s Slow Violence and Stacy Alaimo’s Transcorporeality. These frameworks reveal the lingering, porous exchange of toxins between the landscape and the body, highlighting the urgent need for a transnational approach to ecological and social restoration. Ultimately, the study suggests that true restoration is impossible without acknowledging the shared vulnerability of the land and its people, demanding justice that heals both the landscape and the lineage through the narrative agency of *Dust Child.* The primary aim of this research is to emphasize the environmental and bodily harm caused by chemical warfare and to propose Restorative Justice as a sociological repair mechanism. To this end, the study uncovers the toxic effects of Agent Orange in the lives of the main characters in *Dust Child*. It also substantiates how Restorative Justice enables the repair of socio-political relations in the novel. The legacy of harm and toxicity is documented in a first-person narrative through Testimonial Witnessing. Storytelling eventually leads to the restoration of justice.

The Vietnam War (1955–1975) was a geopolitical conflict between the North and South Communists, supported by the US Army. This intervention also led to a social rift between the Vietnamese and American mixed-race offspring. They were called “Bui-Doi” (Dust of life), the unwanted remains of the war. Under the pretext of politics, the US Army used chemical warfare to destroy the North Korean Communists. Herbicidal Warfare was a tactic used by the U. S military forces during the Vietnam War, which led to Operation Ranch Hand, where nearly 20 million gallons of Rainbow Pesticides, among them the most lethal, Agent Orange. Schuck (1986) mentions, “This toxic dioxin transformed the physical terrains into weapons and civilian bodies into a material site of military harm.” This identifies how chemical warfare inflicts harm on civilians, their surroundings, and their social lives. The herbicide was primarily sprayed to destroy dense forest cover and expose the Viet Cong Army’s hiding places. Waugh (2010) describes this act as “a catastrophic loss of biodiversity,” mentioning that “nearly 20 percent of South Vietnam’s forests were sprayed at least once,” leading to “a massive and enduring transformation of the landscape.” Agent Orange, laced with synthetic dioxin, changed the fertile land of Vietnam into a toxic terrain that altered its ecology and triggered bodily mutations in humans. The toxin penetrated the soil and eventually entered the human body, causing harm through generations. Waugh further notes that “dioxin, the contaminant in Agent Orange, acts as a molecular saboteur, interfering with the most basic biological processes of growth and development in both plants and humans.” Thus, the Vietnam War led to an unprecedented site of ecocide and chemical warfare. Plumwood (2002) and Marchesini (2015) critique this “anthropocentric attitude towards nature and nonhuman life.” The same nature destined to protect human beings was destroyed, uprooted, and became a site of death, war, and damage. The environment still holds the trauma, violence, and toxicity of war, even though the war is over and the hostilities have ended. The narrative of *Dust Child* (2023) by Nguyễn Phan Quế Mai functions as a literary tool to expose the repercussions of such hostilities. The significance of this study lies in the current hostility leading to war in the global context. One crucial instance highlights the current use of chemical warfare. On 5 February 2026, a British Broadcasting Corporation (BBC) News report stated, “Lebanon has accused Israeli aircraft of spraying an agricultural herbicide over southern villages at concentrations authorities described as dangerously high, raising concerns for food and environmental security” (BBC News, 2026). This act raises concerns and awareness about the need to reevaluate the long-term effects of biological warfare on ecosystems and humans. It also acts as a reminder of the material harm caused by Vietnam’s Rainbow Pesticides. Therefore, focusing on such human body interrelations with the environment exposes such delayed violence, toxic embodiment, and psychological trauma related to chemical warfare. The Slow Violence and Transcorporeality frameworks aid this exposition. Trauma and Testimonial Witnessing of war-affected individuals lead to a restorative framework that signals societal efforts such as acknowledgment, forgiveness, testimonial witnessing, and acceptance to address the enduring aftermath of war.

Ancient Vietnamese Literature focused on national integrity and the preservation of cultural heritage against Westernization. However, post-war literature pivoted toward a clear and in-depth understanding of the social repercussions of conflict. Seminal works on Vietnamese post-war fiction include *The Sorrow of War* by Ninh (2005), *When Heaven and Earth Changed Places* by Hayslip and Wurts (1989), and *Novel Without a Name* by Hương (1991). These works exemplify the long-term scars of psychological trauma, the loss of love and innocence, and the moral and ethical complexities of survival that haunt memory within blood-soaked landscapes. Storytelling is an instrument through which the past is remembered, the dead are honored, and historical trauma is reconciled. The protagonist, Kiên, in *The Sorrow of War*, reconciles with his traumatic memory when he finally realizes that he has survived to perform an unnamed heavenly duty. A task that is sacred and noble but secret (Ninh, 2005, p. 45). Writing about war becomes their second chance at life. Collectively, these works convey the importance of bearing witness to the past through narrative voices that may prevent the recurrence of such tragedies. One such work is *Dust Child* (2023) by Nguyễn Phan Quế Mai, which highlights the trauma and displacement of Amerasian children. The catastrophic violence of war through Agent Orange, sexual exploitation of women, and environmental destruction through chemical retaliation. Nguyễn Phan Quế Mai is a renowned poetess, writer, translator, journalist, and social activist among modern-day Vietnamese writers. She is acclaimed for her meticulous blend of realism with vivid imagery, connecting history to fiction. She was born in North Vietnam in 1973 and later moved to the Mekong Delta in Southern Vietnam, where she spent the majority of her life. She later traveled to pursue her Master’s and PhD in Creative Writing from Lancaster University, UK, where she completed the manuscripts of her two novels, *The Mountains Sing* (2020) and *Dust Child* (2023). Her works focus on the sufferings endured during the Vietnam War, fused with historical facts, insisting on peace and communal healing. She explores themes of displacement, cultural identity, and collective memories of Vietnam. Her debut novel, *The Mountains Sing* (2020), is a family saga about three generations of women from the First Indochina War, their struggles during land amendment policies under Communist rule, and their trauma of the Vietnam War. Her second novel, *Dust Child* (2023), explores the delayed effects of Agent Orange on natural landscapes and human bodies. It exposes the emotional complexity of trauma and the repercussions of battle. Nguyễn and Doğan (2025) note in an interview that the novel sheds light on the complex history of the American War in Vietnam and its enduring effects on individuals and society, highlighting voices and stories that are often overlooked.

## 2 Materials and methods

This study employs a qualitative interdisciplinary methodology to analyze Nguyễn Phan Quế Mai’s *Dust Child* (2023) using a systematic interpretive framework. A close textual analysis was conducted to examine the repercussions of chemical exposure in the novel. Using thematic analysis, the study identifies instances of trauma, intergenerational suffering, and social ostracization. As Coser (1963) suggests, literature serves as a unique “social mirror” that reflects the tensions and conflicts inherent in a society. Thus, the article illustrates how Restorative Justice operates as an ethical repair of social relations among characters grappling with the war. The goal is to use literary analysis as a sociological method that mediates the collective experiences of environmental violence and social marginalization. Through the lens of Slow Violence and Restorative Justice, the novel is analyzed to show the toxic implications of chemical warfare and the repair of social relations through the acknowledgment of harm. This study includes photographs recorded at the War Remnants Museum in Ho Chi Minh City, Vietnam. It documents the embodied and intergenerational effects of Agent Orange due to toxic exposure for contextual grounding. These pictures synthesize the narrative data of *Dust Child* to form a corporeal archive in postwar Vietnam.

## 3 Theoretical framework

This study deploys an interdisciplinary theoretical framework for Nguyễn Phan Quế Mai’s *Dust Child* (2023), drawing on Slow Violence, Transcorporeality, and Restorative Justice. They represent the incremental effects of socio-ecological relations in war and the porous exchange of chemicals between the environment and the human body. These aspects of past events are captured by Testimonial Witnessing through the novel’s narrative. This study highlights trauma, violence, and social fragmentation from first-person narratives as a reminder to avoid the use of chemical toxins in war at all costs. As an ethical approach to addressing harm, Restorative Justice is suggested to foster acceptance, acknowledgment, and reconciliation. In unison, these frameworks foster a comprehensive reading of the novel as a locus site where the collapse of nature, materialized toxicity, and ethical response intersect. Consequently, this article contributes to the wider academic discourse on environmental justice and the sociology of law by positioning literary narratives as a pivotal lens for understanding the persistent effects of toxins and the social stigma surrounding warfare.

## 4 Discussion

### 4.1 Unfolding catastrophe: slow violence and its legacies

The theoretical aspect of present-day conflict often portrays “fast violence,” the instant, notorious aftermath of war. Explosions and immediate casualties are shown to abandon the “Slow Violence” that continues in the chemical and biological repercussions. Delayed violence is ignored because it fails to produce the “theatrical quality” that arouses public attention of a sudden explosion. Instead, violence is an attritional force that silently weakens the health of ecosystems and humans. As defined by Nixon (2011), Slow Violence is a “violence that occurs gradually and out of sight, a violence of delayed destruction that is dispersed across time and space, an attritional violence that is typically not viewed as violence at all.” This discussion aims to counter the slow, persistent violence of conflict. It claims that the Vietnam War did not end with the Fall of Saigon in 1975, but instead led to delayed destruction through the environmental and human transference of Agent Orange. In the milieu of history, religion, and sociology, the enduring bequest of toxic legacies is studied, with Davies (2018), that “the past is never truly over but is chemically archived in the soil and the blood of the marginalized.” The significant features of Slow Violence are the invisibility of harm, delayed effects, and institutional avoidance, which give rise to ecological destruction, chronic health effects in humans, and intergenerational seepage. As Deb (2021) explains the delayed effects of gases in nature and human lives, “Bhopal is the embodiment of ‘Slow Violence’ in neoliberalism, not only because the enduring consequences elude political, judicial, and medical discourse but also because the Slow Violence of biosocial and environmental destruction continues to affect marginalized people living in Bhopal.” These instances highlight the “epistemic injustice where the suffering of the poor is rendered illegible to the state” (Boudia and Jas, 2014). In the exposition of Nguyễn Phan Quế Mai’s *Dust Child*, the discourse reframes the “Dust” (*Bụi Đời*) not as the debris of war but as a potent participant in an ongoing chemical encounter. This aligns with Nixon’s highlights of “displacement of time” and “invisible casualties” explain how the characters experience violence inflicted upon them are not mere occurrences but are “the slow-motion residue of a war that refuses to end” (Nixon, 2011). The sociological awareness of this study about Slow Violence lies in shifting the focus from retributive blame to a restorative acknowledgment of this persistent harm. Eventually, Slow Violence in *Dust Child* acts as a material trace of a bloody history that compels an ethical response. It transforms the discourse of past lives into a reality of transcorporeal exchange, in which the toxic environment bears witness alongside the human survivors.

**Figure 1:**
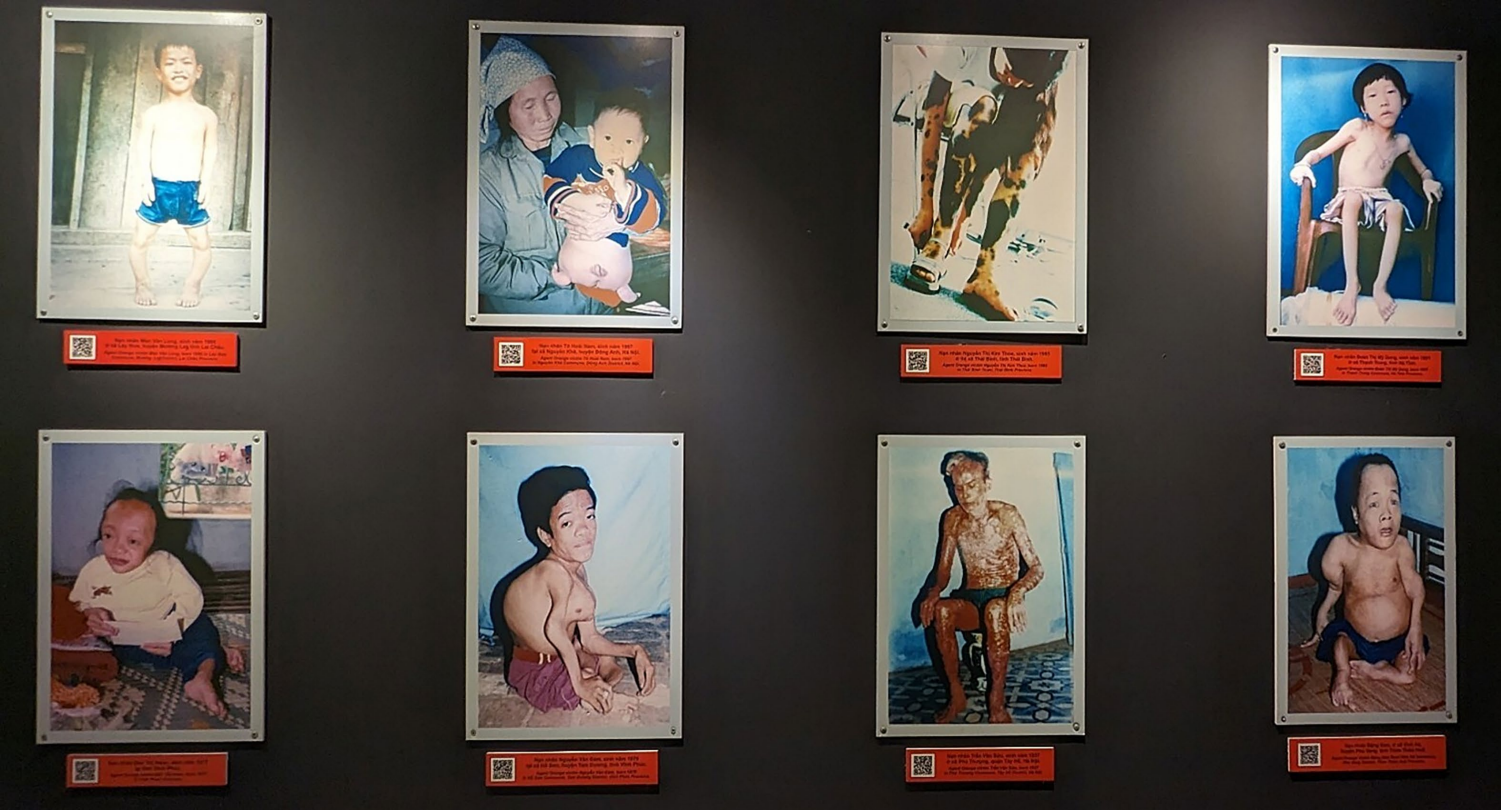
Visual references of congenital conditions and slow violence. A complex collage of eight images showcasing the biological persistence of Agent Orange. These images display crooked limbs, gigantic heads, and skin diseases among post-war generations. These visual references position an empirical counterpart to human-nature enmeshment as developed by Alaimo (2010), complementing the key passage from Nguyễn Phan Quế Mai’s *Dust Child,* confirming the descriptions of children with “missing or crooked limbs” and “heads twice the size of their chest” (Nguyễn, 2023, p. 278). Reprinted with permission from the War Remnants Museum, Ho Chi Minh City, Vietnam.

The non-linear narrative of *Dust Child* starts with Phong, an Amerasian man born to an American father and a Vietnamese mother during the war, searching for his parents, who left him at an orphanage run by Sister Nhã. He represents the childhood trauma and abuse of “dust children” ostracized by Vietnamese society due to their mixed-race origin. Next, the life events after the return of an American Veteran, Daniel Ashland and his wife, Linda to Ho Chi Minh City in 2016 where he was stationed during the Vietnam War in 1969, and flashback story from the past about Trang and Quỳnh, two sisters from Phú Mỹ village of Kien Giang Province in 1969 who leave their rural village due to poverty, destruction of fields, move to the city Saigon as bar girls to cater to the needs of American soldiers. The lives of the main characters are pushed into prolonged poverty, loss of livelihood and identity, and psychological scars across generations due to the war. The line, “War is about violence. The strongest will win, and the weakest will lose. If not strong and violent, one will die” (Rydstrom, 2012). This exhibits how state-composed violence leaves an everlasting impact of death and destruction on weak lives caught in past-war conflict. The novel’s reference to “the war had ended nine years earlier, but the fighting hadn’t stopped” (Nguyễn, 2023, p. 46–47) directly shows how violence extends beyond immediate death, leaving a long-term ecological dispossession. As Nixon (2011) states, “unofficial casualties of war,” showing the incremental destruction. In such armed conflicts imposed by hostilities, nature is the first victim of both fast and Slow Violence: “Outside was nothing but empty fields. Ngân said that American airplanes had been spraying some type of chemical. Since then, all the crops just withered and died” (Nguyễn, 2023, p. 257) exposes the ecocide of war beyond the battlefield, with harm unfolding in land and life. The herbicidal war led to the destruction of approximately 40% of mangrove forests, soil erosion, nutrient runoff along the South Vietnamese coasts, deforestation, and reduced marine life (Yuki, 2025). This instance is highlighted by the vivid description of Phú Mỹ village, which meant “rich and beautiful” before the war, now it has become “stinking jungle” charred by napalm, an explosive petroleum jelly which has burnt the trees and the whole landscape turned into “scorched villages, burnt forests, deserted fields, bomb craters, and scattered bodies of people and animals” (Nguyễn, 2023, p. 238). The collage of visual references in Figure 1 acts as a stark empirical counterpart to the human-nature enmeshment theorized by Alaimo (2010) and the temporal delays characterized by Rob Nixon’s (2011) framework of Slow Violence. Slow Violence is also attributed to poverty, which is portrayed as “The war does not just kill people, it robs our livelihood and destroys nature” (Nguyễn, 2023, p. 257). This symbolizes the destruction of livelihoods through the poisoning of rice fields. The result of this is poverty, which pushes the sisters, Trang and Quỳnh, into a state of sexual exploitation in a bar. State-imposed structural violence is portrayed through the American veteran, Dan, who “recalled the times when he’d accompanied C-123 provider aircraft from Operation Ranch Hand on their spray missions, color-coded drums with orange, pink, purple, blue and white stripes at Bien Hoa and Tan Son Nhut airports, called Rainbow pesticides to kill plants and people” (Nguyễn, 2023, p. 279). This explicitly refers to the invisible visceral violence under the pretext of sanitized labels called “pesticides.” It manifests poison sown in the Vietnamese soil that would slowly destroy the inhabitants of the land. Thus, the events of Slow Violence testify to the delayed effects of chemical warfare, which leaves everlasting damage to the landscape and characters in the novel.

Slow Violence is imposed in the form of poverty and survival of Vietnamese sisters and socio-economic abandonment of Phong, the “Bui Doi – Dust of Life” (Nguyễn, 2023, p. 114). Their lives have an eternal state of poverty and desperate struggle for survival: “Quỳnh scraped and scraped the rice pot with a spoon, but there were no grains left” (Nguyễn, 2023, p. 21). This vividly highlights the economic drain as a form of Slow Violence due to the destruction of their rice fields by chemical warfare, thrusting them into a life of sexual violence: “Quỳnh was gagged, tied to a bed and hit by an American soldier” (Nguyễn, 2023, p. 220). The portrayal of how women become embodiments of gendered violence and sexual exploitation through power structures is often discredited. Next, the lives of Amerasians are adversely affected by Slow Violence through their abandonment, displacement, and abuse, as they were the by-products of a war that ended years ago. Phong recalls a childhood memory when he was wandering the streets for food, who knelt, shivered, and begged for food at merciful hands, and “felt mortified” (Nguyễn, 2023, p. 102). These lines are a clear indication that they are victims of Slow Violence, ‘dust children’ who are the unwanted dust of war. Therefore, through the delayed and cumulative effects of chemical warfare in *Dust Child*, the study argues that the notion of war is not temporary and immediate but rather a prolonged aspect of suffering. Slow Violence serves as a medium to establish the fact that sustained violence, loss, and trauma exist long after the war has ended.

### 4.2 Toxic bodies, porous boundaries: Transcorporeality

**Figure 2:**
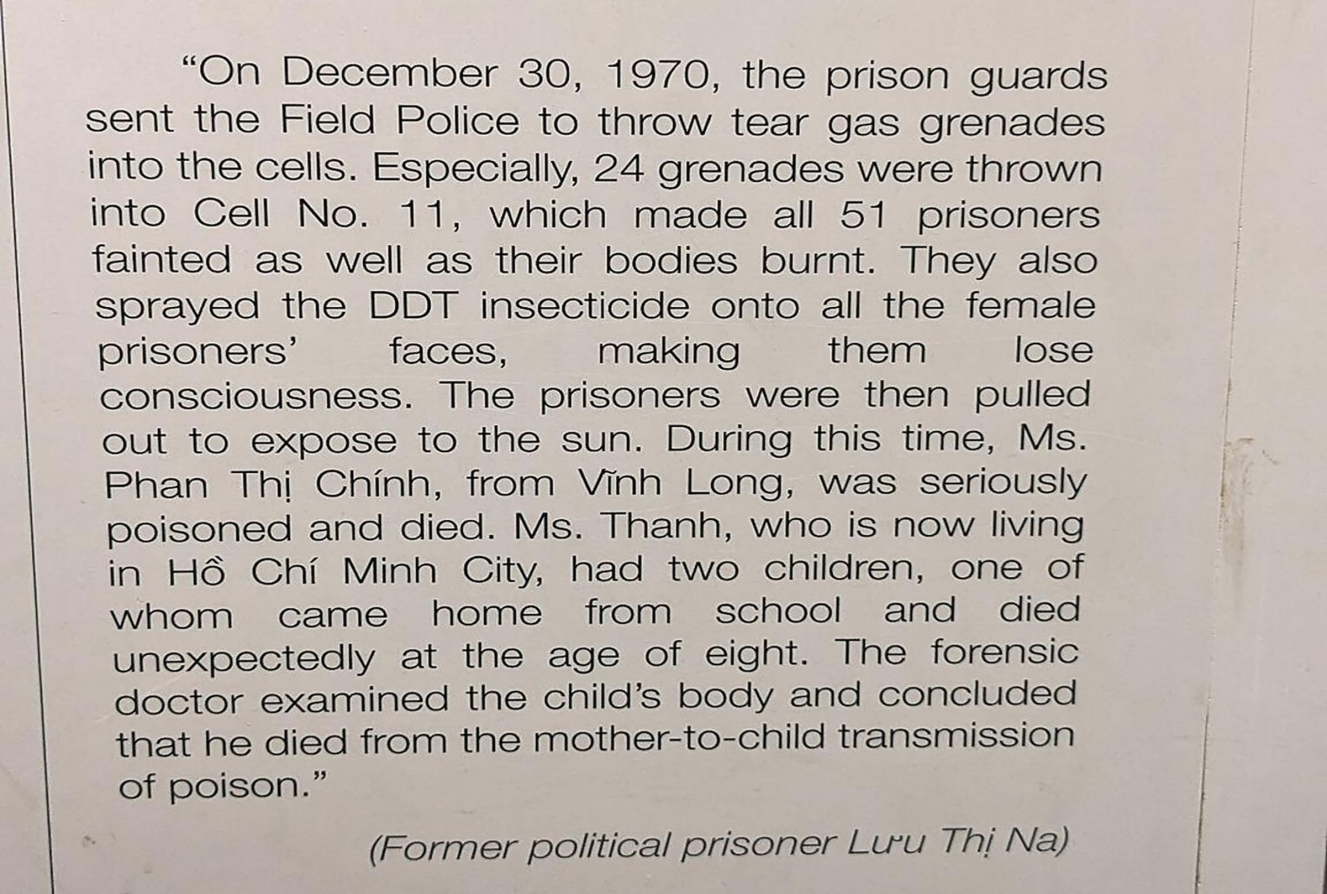
Material corroboration of mother–fetus chemical transmission. Historical documents from the War Remnants Museum, Ho Chi Minh City, Vietnam, highlight the use of DDT, a persistent organic pollutant used for malaria control, combined with Agent Orange. The photograph documents the exposure of a female Vietnamese prisoner to chemical spraying and the death of her eight-year-old child years later. The forensic examination concluded that the cause of death was mother–child poison transmission due to the chemical. This case serves as an empirical case study of Transcorporeality and the porous permeation of the human body within a toxic environment contaminated by Agent Orange. Reprinted with permission from the War Remnants Museum, Ho Chi Minh City, Vietnam.

The temporal linkage of Slow Violence to the material phase of the environment and humans is studied through the lens of Stacy Alaimo’s theory of Transcorporeality (2010). It posits that “the human is always intermeshed with the more-than-human world” (Alaimo, 2010), referring to the human body not as a sealed entity but a porous site of constant exchange of matter and energy with its toxic surroundings. The superficial partition between the natural habitat of Vietnam and the Vietnamese people exposes how the environment is poisoned by dioxin, Agent Orange, which travels through soil and water to enter the human bloodline. Yuki (2025) states, “It has not yet occurred to people that a woman’s womb and the sea are the same tide. Where the poison was injected directly,” through the reading of Ishimure (2004) memoir *Paradise in the Sea of Sorrow: Our Minamata Disease* (1990). These lines mention the visceral and biological link between humans and the environment in the aftermath of mercury poisoning of the sea in pregnant women. From a sociological perspective, the past-present connection is described as ‘material entanglement’ where the body retains what the state neglects. Oppermann (2013) states, “Transcorporeality allows us to see the body as a site where distant industrial histories become immediate biological realities.” In this context, *Dust Child* by Nguyễn Phan Quế Mai is interpreted through the framework of Transcorporeality, which reveals that the dust children are the literal embodiment of this biological reality. Somatic memories are carried in the DNA, bloodlines, and physical disfigurements of the novel’s characters. Toxic substances from chemical warfare are inseparable from humans and the environment. Agent Orange transforms from a historical weapon to a permanent biological residue of war. This article leverages transcorporeal reading to foreground the biological testimony of Amerasian children, whose existence functions as material evidence of a war that refuses to be eradicated. The narrative of *Dust Child* establishes the internal effects of war permeating deeply, blurring the boundary between environment and body, and transcending external time and factors. The environmental seepage of toxins is highlighted in “under the green shades, shades of black and brown: scorched villages, burnt forests, deserted fields, bomb craters, and scattered bodies of people and animals” (Nguyễn, 2023, p. 238). This signals not just an ecological collapse but also the residual toxicity permeating nature that sustains life. Several instances of environmental violence are pointed out throughout the novel. Dan recalls the times when he accompanied C-123 provider aircraft from Operation Ranch Hand. On their spray missions, many color-coded drums of Agent Orange, Agent Green, Agent Pink, Agent White, and Agent Blue: Rainbow pesticides were deployed (Nguyễn, 2023, p. 279). The study presents this defining moment as compelling evidence to record the transcorporeal violence deliberately imposed on the Vietnamese landscape. This chemical harm led to toxic exposure in nature through soil, water, and ecosystems. This provides clear evidence that the environment is no longer a passive backdrop but an active force through which violence disperses and persists in the human body. The internalized violence of war can be inferred by the physical injuries sustained by Trang’s father: “Pieces of shrapnel were still buried deep in his lungs” (Nguyễn, 2023, p. 30). This suggests that war is not only a part of memory alone, but it is a lived, psychological reality. Likewise, Trang’s own experience of unexplained bodily suffering can be observed from the lines, “Her shoulders ached, four rice seasons ago, when she’d started working full time, she’d thought the constant pains in her body were caused by a serious illness, probably cancer” (Nguyễn, 2023, p. 19–20). The lines, “Around him, people collapsed, dying from the different illnesses that swept through the camp” (Nguyễn, 2023, p. 50), allude to the permeability of bodily matter and toxins like Agent Orange. Historically, the spots of Trang’s village, Phú Mỹ Village from Kien Giang (A Luoi valley) province, and Phong’s reeducation camp in Lam Dong were heavily sprayed and contaminated by dioxin (Hatfield Consultants, 1998). Both the cases of Trang and people from the camp have the common factor of farming in a field, which was symbolically the” battleground of war.” This reinforces the high possibility of cancer and deaths due to the presence of toxins in the DNA of the people caused by exchange through soil, water, and food chains. The material exchange of intergenerational permeation of chemicals is evidenced by visual archives of dichlorodiphenyltrichloroethane (DTT), a persistent organic pollutant. As seen in Figure 2, the archival documentation of a female Vietnamese prisoner exposed to a lethal combination of DDT and Agent Orange underscores what Alaimo (2010) identifies as the radical vulnerability of the human body to its toxic surroundings. Tuana (2008, p. 193) reveals in her study on “viscous porosity” that there is no stark difference between the physical self and natural environment; rather, it is a state of perpetual transformation within the environment. These moments explain that the viscous human body and environment do not just endure war externally but absorb the immediate effects of chemicals, sustain them, and become a prolonged agency of contamination and permeation. The bodily entanglement and persistent residual toxicity are further strengthened by the current reference, where Dan and Linda saw homeless people and beggars. They had disfigured faces and missing limbs after fleeing from the heavily bombed and dioxin-contaminated areas of the Central and Mekong Delta regions (Nguyễn, 2023, p. 176). In one instance, Dan and Linda visit an orphanage for children affected by Agent Orange. The lines, “gigantic heads, missing or crooked limbs, some children unable to speak and a young girl whose head was twice the size of her chest” (Nguyễn, 2023, p. 278), powerfully present the bodily deformities sustained by toxic exposure and bioaccumulation. This depiction is supported by empirical archives documenting the material consequences of chemical exposure. Sensory feelings further show this permeability of the body as Trang notes about Dan when he returns from war, “His skin smelled different. It smelled of death and anger” (Nguyễn, 2023, p. 218), referring to how violence is not only witnessed but is absorbed by the magnitude of skin, breath, and perception. In this way, human bodies become a medium through which war circulates, embedded in both physical and sensory form. With reference to these events of destruction and material harm, the study sheds light on how poisonous chemicals infiltrate an ecosystem and directly and indirectly enter the food chain. Hence, the study calls for a resolution to stop the use of toxic chemicals in warfare.

### 4.3 Witnessing trauma: testimony and memory

The lethal consequences of toxins are often neglected by dominant political narratives, leading to an epistemic injustice. Through the mapping of Felman and Laub’s (1992) concept of the “crisis of witnessing,” restoration and repair are facilitated. Psychologically, Trauma is a slowly and invisibly induced condition that affects survivors who carry the burden of warfare. Caruth (1996) argues that “the moving and sorrowful voice that cries out, a voice that is paradoxically released through the wound” concerns the process of trauma through repetition of a painful experience that was not fully processed when it occurred first. This pain and trauma can be shared and voiced to help heal the wound. In the past, storytelling practices were used to share these traumatic experiences. The Fambul Tok system in West Africa characterizes traditional community healing, helping victims and lawbreakers coexist in the same society. As Hoffman (2014) mentions, “Fambul Tok’s primary aim is to facilitate community-led reconciliation by providing a space for victims and perpetrators to come together. The purpose is to tell their stories, acknowledge harms, and begin the process of relational repair through traditional, bonfire-lit ceremonies that emphasize forgiveness over retribution.” These customs highlight how storytelling can serve as an act of remembrance, reclaiming a silenced history. This study sheds light on these physical and psychological scars of war that form the “testimony” of the characters in the novel. Their bodies bear witness to a catastrophe forgotten by the world. As Felman and Laub (1992) propose, “testimony is not simply a statement of facts but a performative act that requires a listener to fulfill the process of witness.” Confrontation with biological archives changes the aspect of “Dust” from a metaphor of abandonment into a collective healing by common acknowledgment and listening. As Wallace (2016) attests, this action has become the primary method for the restoration of justice, establishing the truth of the dust children by their testimony and recognition of the crime by the perpetrators globally.

The embodiment of trauma and the psychological persistence of war is a powerful message conveyed through this study. Unprocessed trauma is preserved in the body and mind of both victims and offenders in conflict. The traumatization is preserved from past to present through memory and fear, disturbing the temporal existence of reality. War veterans like Dan and Thanh’s father are the primary embodiment of trauma who absorb the weight of duty to their nation, power structures that operate them. They sustain the impact of violence, the terror of war, anxiety from brutal actions, and the curse of carrying the guilt of their wrongdoings eternally. Their post-traumatic stress disorder (PTSD) condition can be captured through their nightmares: “In Mekong Delta, a man ran behind him and stabbed him shouting, “Return my wife and children to me” the same farmer kneeling and howling outside a burning home” (Nguyễn, 2023, p. 63), “He stared at the bowl of tomato soup, cupped his palm against his mouth, and vomited. do not cook anything red, blood, blood, blood he’d seen or that he’d caused to spill” (Nguyễn, 2023, p. 218) and “He cannot be in a room with a ceiling fan, the spinning blades would terrorize him and remind him of American helicopters, they dropped soldiers to hunt him, twice chased and tried to shoot” (Nguyễn, 2023, p. 248). These lines provide evidence that warfare does not end with material annihilation. Instead, it incessantly invades the human psyche as a dormant force through involuntary memory, sensory triggers, and corporeal fear.

The character of Phong represents the societal subjugation and marginalization of Amerasian children whose childhood and adulthood are shaped by racial and social ostracism. He is called a half-breed, dust of life, bastard, Black American imperialist, child of the enemy (Nguyễn, 2023, p. 1). His pain and shame are depicted in an instance where Phong rubbed his skin until he bled profusely to erase his black identity from his body (Nguyễn, 2023, p. 2), Vietnamese call them, “Bui Doi – dust of life, mostly poor, no school, and finding jobs is difficult (Nguyễn, 2023, p. 114). This harsh reality is highlighted in an interview by the author, Nguyễn: “Their trauma often goes unrecognized, brushed aside by both Vietnamese and American societies” (Nguyễn and Doğan, 2025). As Caruth mentions, “The experience of the soldier faced with sudden and massive death around him suffers this sight in a numbed state, only to relive it later on in repeated nightmares, is a central and recurring image of trauma in our century” (1991). These examples convey Amerasian children as a painful and shameful residue of war. To voice the silenced trauma witnessed by these people, the investigation positions the act of storytelling, literature, and testimony as a key factor through which trauma is communicated and acknowledged. Dan’s attachment to the book *The Sorrow of War* (Ng, 2014) is an effort to humanize his former enemy, Kien from North Vietnam, and becomes an act of ethical recognition through which he regains his own humanity (Nguyễn, 2023, p. 15). Likewise, for the Vietnamese sisters Trang and Quỳnh, writing and storytelling create a safe space from the terror of war and violence, even though there was no truth in the letters…writing had enabled them to escape the horror” (Nguyễn, 2023, p. 331). These acts do not erase trauma but transform it into a narrative, allowing experiences that would otherwise remain silenced to be expressed and shared. The analysis suggests that witnessing is not merely recounting events. It is also an ethical act that confronts denial and restores visibility to marginalized lives and acknowledgment. In doing so, *Dust Child* bridges the material persistence of war with its narrative articulation, preparing the ground for a broader demand for recognition and justice. Storytelling is identified as an ethical act of witnessing: Dan’s engagement with *The Sorrow of War* allows him to “humanize” the enemy and “regain his own” humanity (Nguyễn, 2023, p. 15), while for Trang and Quỳnh, writing becomes a mode of survival, where “writing had enabled them to escape the horror” (Nguyễn, 2023, p. 331). These moments in the novel transform trauma into testimony, enabling silenced experiences to be narrated, recognized, and carried forward as part of a collective memory and acknowledgment. The truth of the testimonial witness is manifested through an interview with the author, Nguyễn, by Maria Dogan, about her sound belief in “the power of literature to foster empathy and cultural recognition (Nguyễn and Doğan, 2025). According to these perspectives, witnessing is not simply recounting events but an ethical act of justice that confronts denial and sheds light on marginalized lives. Hence, justice is not retributive, to punish, retaliate, and seek revenge; rather, it is Restorative Justice, where testimony creates an ideal surrounding for healing and acknowledgment. As the author explains, “Engaging with their stories was profoundly moving - it allowed me to see beyond childhood perceptions of them as enemies and understand their humanity and enduring trauma.” Thus, Testimonial witnessing proves to be a successful tool for mitigating justice for both Vietnamese and Americans.

### 4.4 From testimony to justice: restorative possibilities

According to the Retributive Justice model, a criminal offense committed by a person against the law warrants proportionate punishment and exclusion from society. However, according to the Stanford Encyclopedia of Philosophy, the model can be an eye for an eye and morally repugnant because it views pain as a positive solution (Walen, 2021). Current debates have replaced retributive justice with Restorative Justice in the criminal system to provide opportunities for offenders to take accountability for the harm, facilitate healing, and support their inclusion in society. This restorative movement rejects the state-centric “monarch’s program of domination” (Braithwaite, 1989, p. 323) and instead prioritizes the repair of “wrongs done to another person” and the land. In a positive sense, Restorative Justice aims for “the healing of the wound and the restoration of peace and order. It seeks to strengthen and build harmonious social structures, remove exploitative practices and attitudes, and knit the warring states back into the global community” (Tierney, 2006, p. 82); thus, it focuses on the emotional union of offenders and victims in society. The Truth and Reconciliation Commission (TRC) is a historical initiative established in 1995 in Cape Town, South Africa, after the abolition of Apartheid (1984–1994). The commission was a court-like Restorative Justice enforcement mechanism in which victims were invited to testify about the breaches of human rights, and perpetrators sought absolution by giving full public recognition of the harm. As Tutu (1999) stated, “The central purpose of the Commission was to promote national unity and reconciliation in a spirit of understanding which transcends the conflicts and divisions of the past by witnessing the truth.” Similarly, the analysis of Nguyễn Phan Quế Mai’s *Dust Child* makes international attempts to restore peace and reconciliation in war-torn nations. The study transforms the toxic legacy of Agent Orange into a narrative of common acknowledgment. The National Sorry Day by the Australian government to publicly acknowledge the removal of Aboriginal children between 1910 and 1970 establishes acceptance of both Indigenous and non-Indigenous groups. Parallel to this, Quế Mai’s *Dust Child* fulfills the act of “closing the gap” between Americans and Vietnamese affected by war. This transforms memory into testimony, asserting that justice starts with acknowledgment and recognition (Manne, 2001).

Recognition of harm and moral responsibility is (the) first step toward restoring justice between communities that were former enemies without the interference of law. Dan’s acceptance of his past mistakes, irresponsible actions, and his willingness to take responsibility as a father are established in the novel (Nguyễn, 2023, p. 287). It is evident from the references “we’re here to offer our apologies, and to make amends” (Nguyễn, 2023, p. 249), “providing financial support for Amerasian Phong to become a carpenter” (Nguyễn, 2023, p. 303), and his charity organization to provide funds to support psychological support for Agent Orange survivors (p. 326). All these repair actions by Dan indicate the purgative soul of Dan, recognizing his past guilt and shame. He becomes an instrument to revive and bring a difference to the lives of Agent Orange survivors and the Vietnamese. This recognition is not limited to individual guilt but a shared suffering by both sides and an act by Thanh, who unites Dan with his father, a North Communist soldier, “He hurried to the altar, ignited a match, lit sticks of incense, Dan stood up, his head bent; he prayed for innocent lives lost, for bleeding wounds to heal, for those who had been wronged to be able to forgive” (Nguyễn, 2023, p. 249). The compelling effort by a “child of war” forms a human bridge enabling forgiveness, acceptance, and acknowledgment of harm in war-torn Vietnam. This leads to the achievement of Restorative Justice, which resonates with Braithwaite’s lines: “Restorative justice is about creating spaces were not only offenders, but other concerned citizens as well, will find it safe to take active responsibility for righting the wrong” (Braithwaite, 2002a, 2002b). Equal significance is given to socially ostracized Amerasian children like Phong and others who have endured the Slow Violence and embodied trauma of war. Sister Nhã from the orphanage says to Phong, “You are the best thing that ever happened to me” (Nguyễn, 2023, p. 46). She transforms his life beautifully through the love and affirmation he always lacked from society. Healing is also enabled through the final union of Phong with his birth mother, Quỳnh, who seeks his forgiveness: “Phong leaned toward her, saying, “Cảm ơn Ma.” He thanked her with not just his word, but also his smile (Nguyễn, 2023, p. 324). All these acts reinforce that collective healing and rebuilding communities are possible through funding orphanages, education, and future generations. This enables them to live in a “peaceful world where humans were kind to other humans, so that no one needed to live with regret and sorrow” (Nguyễn, 2023, p. 332). Such spaces become a haven for the restoration of justice through love, caregiving, forgiveness, identity, and belonging.

In a historical context, several initiatives were made by the US government to repair the harm caused by Agent Orange as a step toward Restorative Justice. The Da Nang airbase cleanup project has been initiated. This is an effort by the governments of the US and Vietnam to collaborate in 2018 to clean up approximately 90,000 cubic meters of soil contaminated with Agent Orange (Tuoi Tre News, 2019) accounts, the Bien Hoa airbase cleanup project has been ongoing since 2019 to clean up the largest remaining dioxin hotspot in Vietnam. Additionally, the US government funds assistance programs for the victims of Agent Orange to offer rehabilitation, prosthetics, and social inclusion programs (Martin, 2022). These remediation efforts attempt to use material and social forms of Restorative Justice to address the enduring ecological and biological consequences of chemical warfare. Therefore, this study endorses Restorative Justice as a healing mechanism for the ecological and biological harm caused by war. It leads to a sociological reality of peace, reconciliation, and empathy, restoring humanity in a world fractured by war.

## 5 Conclusion

The chemical defoliant Agent Orange operates as a toxic archive that cannot be limited to the past but continues to harm through generations and ecological collapse. It is a persistent toxin that gradually poisons landscapes, bodies, and genealogies over time, where lived experiences are registered through storytelling and accountability. Therefore, this transcorporeal enmeshment of material exchange reminds both victims and perpetrators that forgiveness is not forgetfulness but reconciliation. Transcorporeality becomes a medium, creating awareness of the destructive effects on ecology and mankind. Testimonial Witnessing facilitates communication between the affected and offenders and results in Restorative Justice between them. Simultaneously, the socially traumatized and ostracized Amerasians have a platform to testify to the truth, unburden their trauma, and gain acceptance from society. Justice is restored not by punitive measures but through the purging acts of offenders, seeking forgiveness, taking responsibility, acknowledging harm, and, eventually, forgiveness and acceptance by the victims. By utilizing material embodiment and social repair in parallel, the article directs toward a social lens of justice that magnifies the consequences of warfare on Vietnamese landscapes, civilians, children of exposure, and marginalized communities. Thus, the literature becomes a platform for testimony. By intertwining memory, trauma, and voice with “Bui Doi - dust of life,” narrative agency is given to those historically marginalized. Fiction serves as a hegemonic force that voices suffering and trauma, and demands justice. Agent Orange is politicized not as a past ordeal but as a concealed force still present. The exposition of *Dust Child* establishes how warfare influences a geographical location, infiltrates bodies, alters the psychological realm, and thus impacts the socio-politics of a country as a whole. The dust children challenge political and cultural accounts that attempt to erase their history. Phong’s life journey from dust to life metaphorically symbolizes the pain, shame, and abandonment experienced by all Amerasian children. People who are “considered children of the enemy” (Nguyễn, 2023, p. 1) rise above the dirt through hard work and claim a space of their own in society. This discourse also uplifts the Amerasian community, raising above the physical harm and racial and social discrimination in Vietnam that leads to a state of change and acceptance.

Thus, Reconciliation and Communal healing in *Dust Child* become an alternative solution to resolve the evil roots of war, such as terror, racial violence, and suppression. Collectively, Dan’s repentance and Phong and Quynh’s forgiveness affirm that past actions can never be buried; they continually resurface to destabilize the present and shape the future. Nguyễn redefines dust children as bearers of societal resistance, reminding us that justice begins with the refusal to be silent. This peace between communities is stated in, “Mr. Dan and Mrs. Linda are our family, for the many things they have done for us, and we for them. But more than that, we share a common history that bonds us together stronger than any blood ties” (Nguyễn, 2023, p. 327). The article echoes these lines about the transformation of offenders from enemies to extended family members, thereby fulfilling the sociological aim of communal healing. Regardless of the violence and dust-filled warfare, the end of their life journey shares a common history with a new foundation of peace.

## Data Availability

The original contributions presented in the study are included in the article/supplementary material, further inquiries can be directed to the corresponding authors.
